# Supplementary figures and images for: Microglial deficiency in the ATRX chromatin remodeler elicits a viral mimicry immune response that impacts neuronal function and behavior
Source: PLoS Biol. 2025 Sep 12;23(9):e3002659. doi: 10.1371/journal.pbio.3002659 (PMC12445524; doi:10.1371/journal.pbio.3002659)

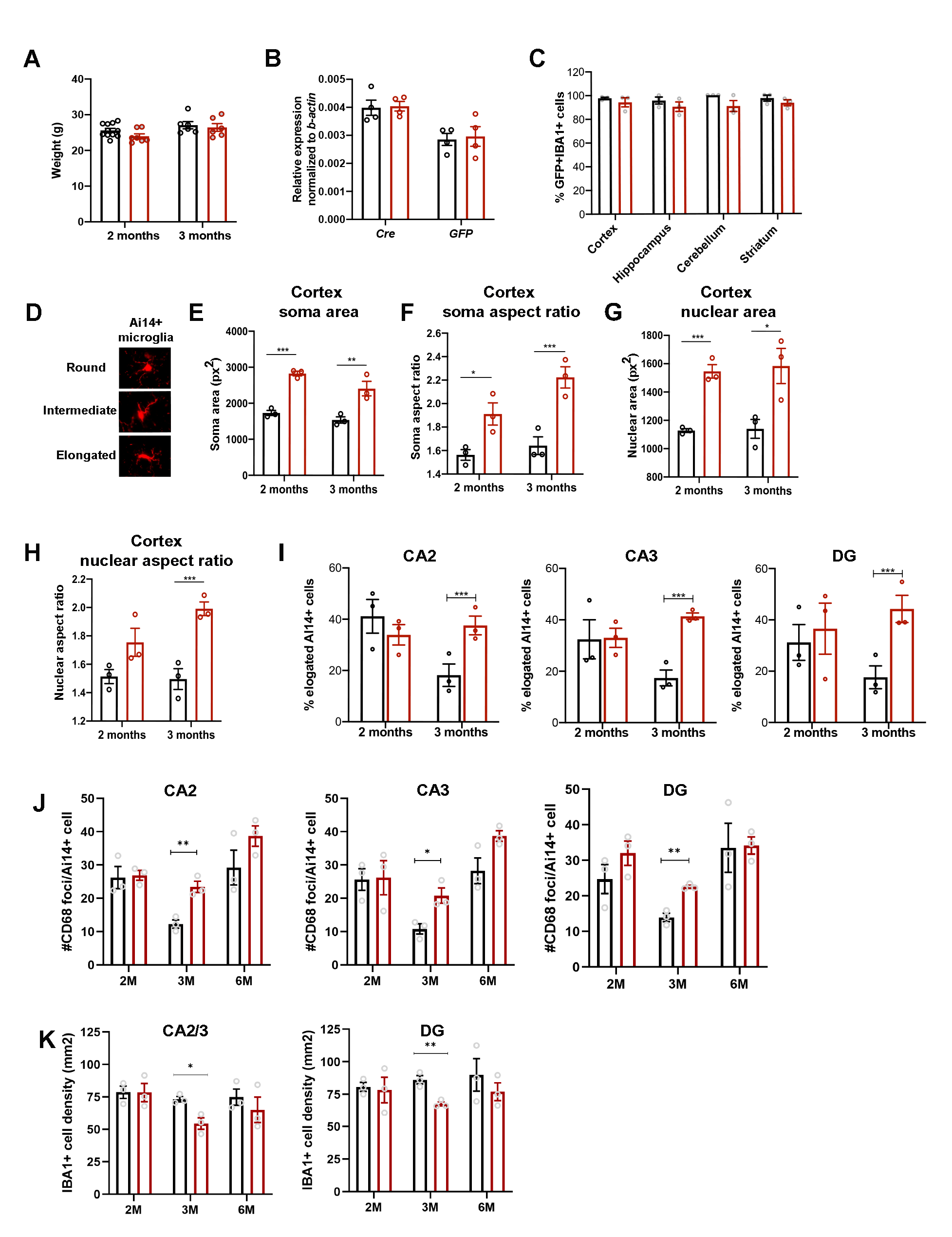

Supplement: S1 Fig — (A) Weight of 2- and 3-month-old control and ATRX miKO mice (2 months, CTL n = 11, ATRX miKO n = 7, p = 0.080; 3 months, CTL n = 6, ATRX miKO n = 6, p = 0.679 Student T test). (B) RT-qPCR of Cre and Sun1GFP transcripts in 2-month-old control and ATRX miKO mice (n = 4 each genotype, Sun1GFP p = 0.871; Cre p = 0.804, Student T test). Results were normalized to beta-actin transcript levels. (C) Quantification of immunofluorescence staining of Sun1GFP and IBA1 reveals >95% Cre expression in control and ATRX miKO mice across different brain regions (n = 3 each genotype, cortex p = 0.423, hippocampus p = 0.351, cerebellum p = 0.119, striatum p = 0.327). (D) Representative images of Ai14+ cells with round, medium, and elongated soma. (E, F) Quantification of soma area and aspect ratio in the cortex at 2- and 3-months of age (n = 3 each genotype, soma area 2 months p = 0.0003, 3 months p = 0.016; aspect ratio 2 months p = 0.029, 3 months p = 0.007, Student T test). (G, H) Quantification of nuclear area and aspect ratio in the cortex at 2- and 3-months (n = 3 each genotype, nuclear area 2 months p = 0.001, 3 months p = 0.034; aspect ratio 2 months p = 0.0937, 3 months p = 0.005, Student T test). (I) Quantification of elongated soma of microglia in hippocampal CA2, CA3, and DG of 2- and 3-months-old mice (n = 3 each genotype, CA2 elongated p = 0.027; CA3 elongated p = 0.002; DG elongated p = 0.018, Student T test). (J) Quantification of CD68 foci per Ai14-labeled microglia in hippocampal CA2, CA3, and DG of 2-, 3-, and 6-month-old mice (n = 3 each genotype, 2 months CA2 p = 0.861, 3 months CA2 p = 0.006, 6 months CA2 p = 0.193; 2 months CA3 p = 0.931, 3 months CA3 p = 0.023; 6 months CA3 p = 0.067; 2 months DG p = 0.243, 3 months DG p = 0.002, 6 months DG p = 0.935, Student T test). (K) Microglia density in hippocampal CA2/3 and DG of 2-, 3-, and 6-month-old control and ATRX miKO mice (n = 3 each genotype, CA2/3 2 months p = 0.975, 3 months p = 0.018, 6 months p = 0.451; DG 2 [file pbio.3002659.s001.tif]

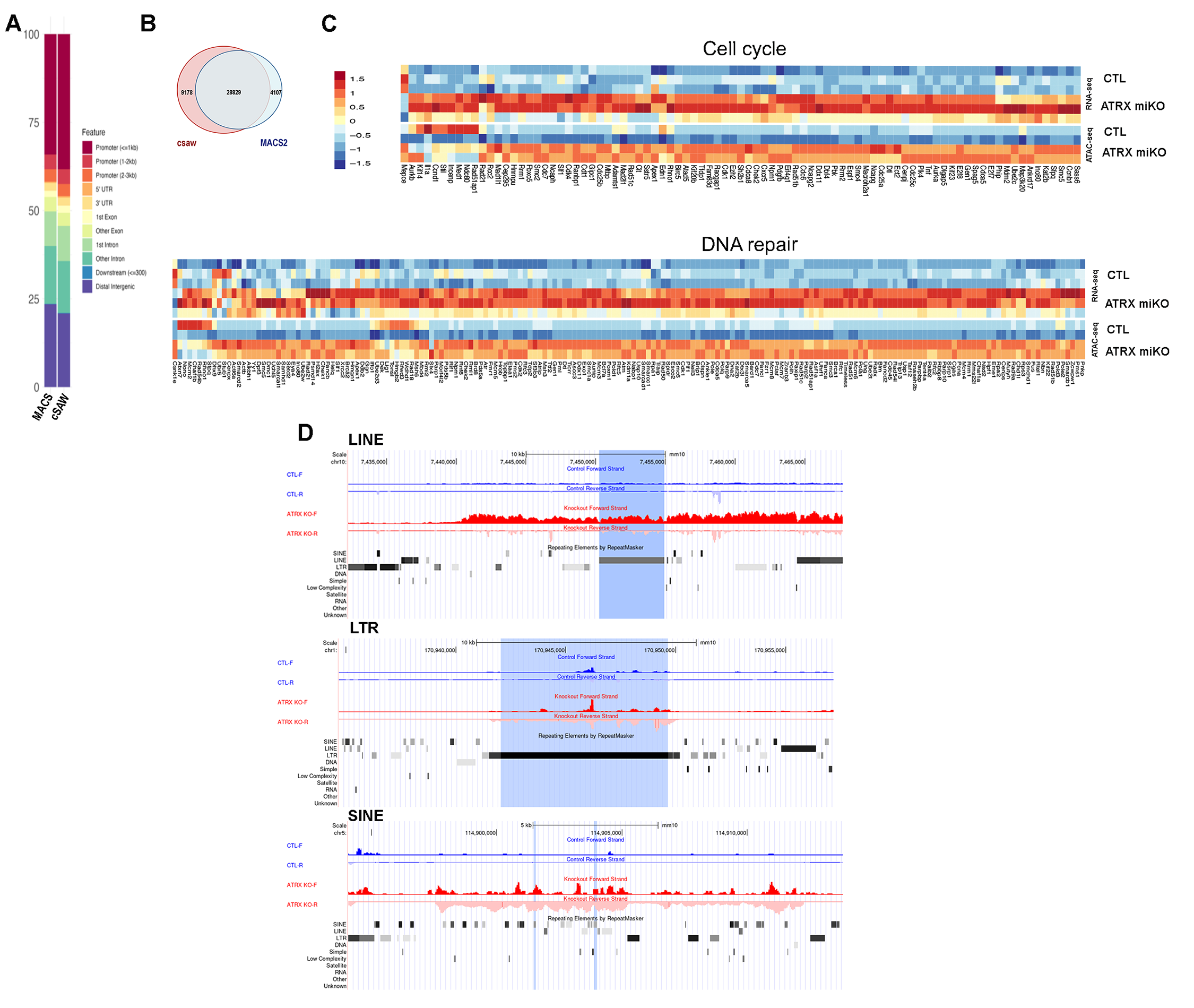

Supplement: S2 Fig — (A) Genomic distribution of DARs called by csaw and MACS. (B) Overlap of DARs called by csaw and MACS. (C) Heatmaps representing an association between gene expression and chromatin accessibility for cell cycle and DNA repair pathway genes. The z-score was computed from the RNA expression or ATAC-seq signals. (D) Macro view of UCSC tracks for example LINE, LTR, and SINE from Fig 4G. (TIF) [file pbio.3002659.s002.tif]

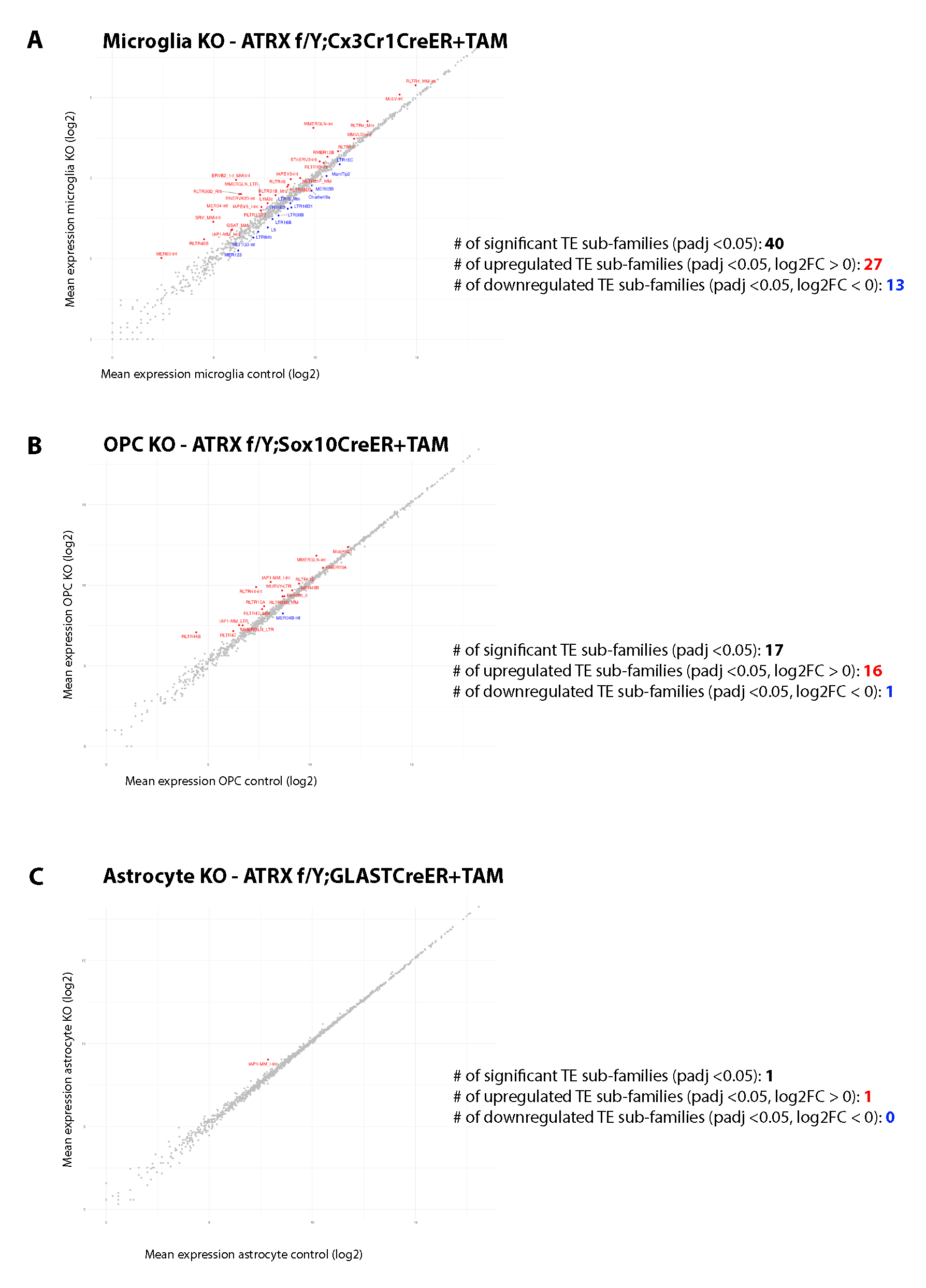

Supplement: S3 Fig — Scatterplots of transposable element family expression differences between control and ATRX KO in (A) microglia, (B) oligodendrocyte precursor cells (OPCs), and (C) astrocytes. Significantly upregulated TE subfamiles are indicated in red and downregulated TE subfamiles are indicated in blue. TE subfamilies not displaying significant change between control and KO cells are shown in gray. (TIF) [file pbio.3002659.s003.tif]

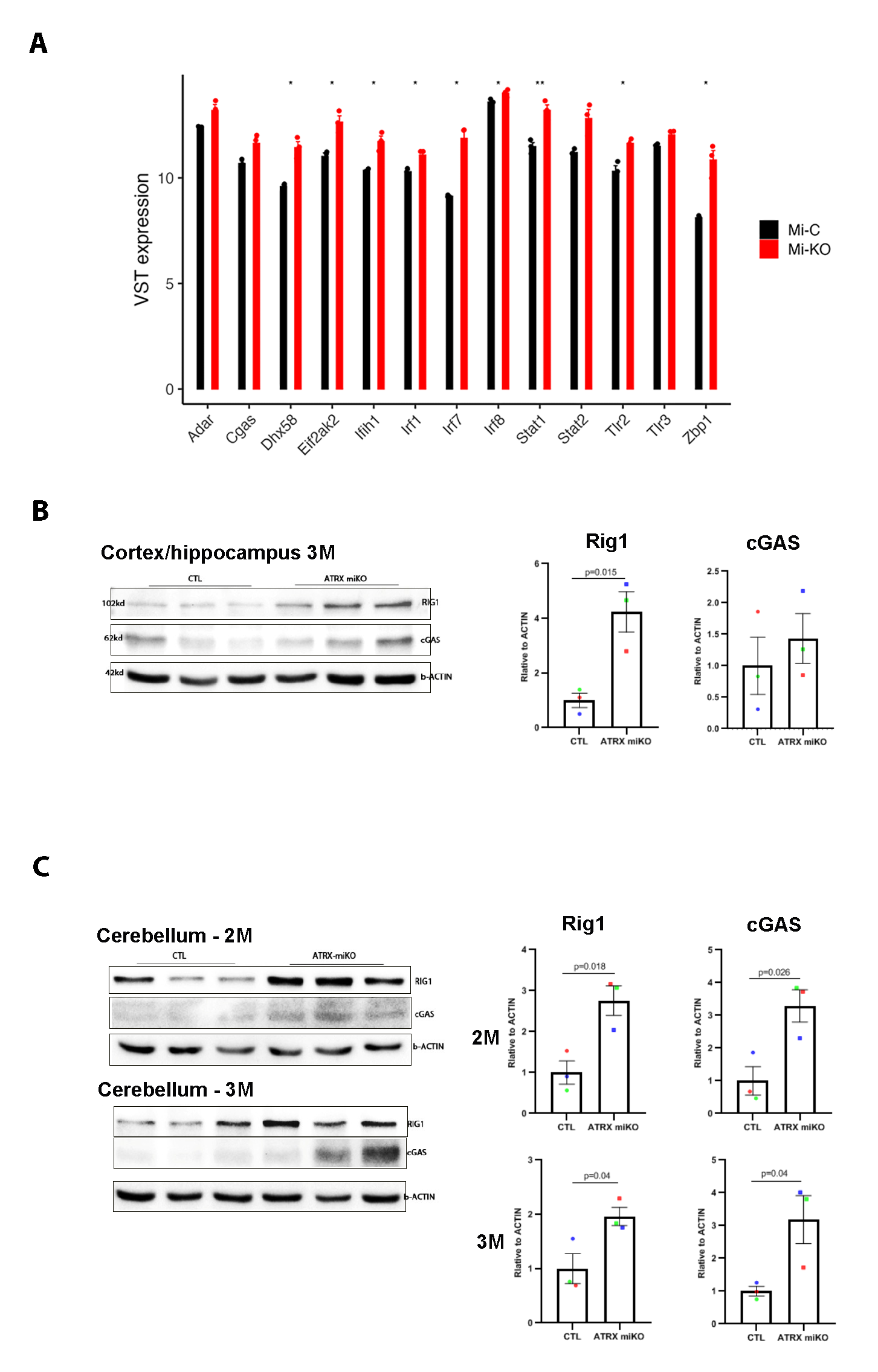

Supplement: S4 Fig — (A) Expression of genes in the RIG1 and cGAS pathways. Variance stabilizing transformation (VST) counts from RNA-seq data are shown. n = 3 each genotype. (B) Western blot analysis of RIG1 and cGAS in control and Atrx miKO cortex and hippocampus at 3 months. Quantification is shown in graphs on the right (n = 3 each genotype). (C) Western blot analysis of RIG1 and cGAS in control and Atrx miKO cerebellum at 2 and 3 months. Error bars represent ±SEM. n = 3 each genotype. Unpaired Student T test. The data underlying this figure can be found in the S1 Data file. (TIF) [file pbio.3002659.s004.tif]

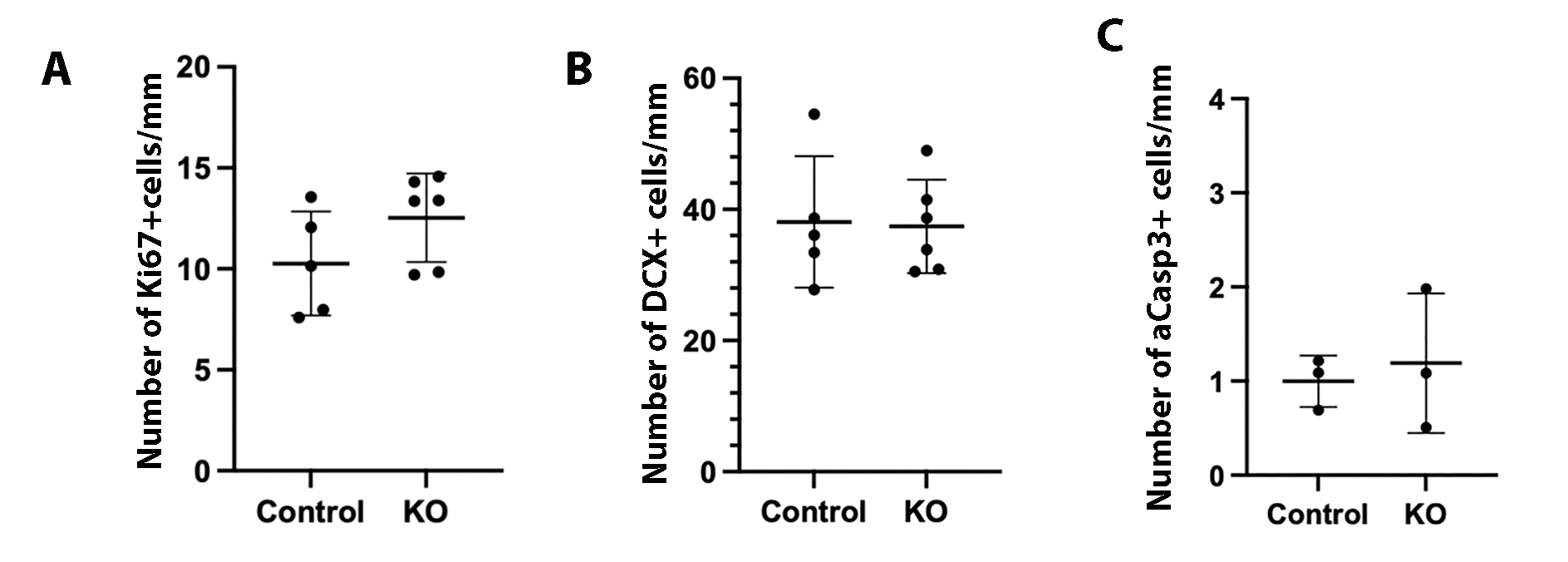

Supplement: S5 Fig — Quantification of (A) Ki67+ proliferating cells and (B) DCX+ differentiating cells in the dentate gyrus of control and ATRX miKO mice (n = 5 control and n = 6 KO mice). (C) Immunofluorescence staining of dentate gyrus for activated caspase 3 reveals no difference in apoptosis between control and ATRX miKO mice (n = 3 each genotype). The data underlying this figure can be found in the S1 Data file. (TIF) [file pbio.3002659.s005.tif]

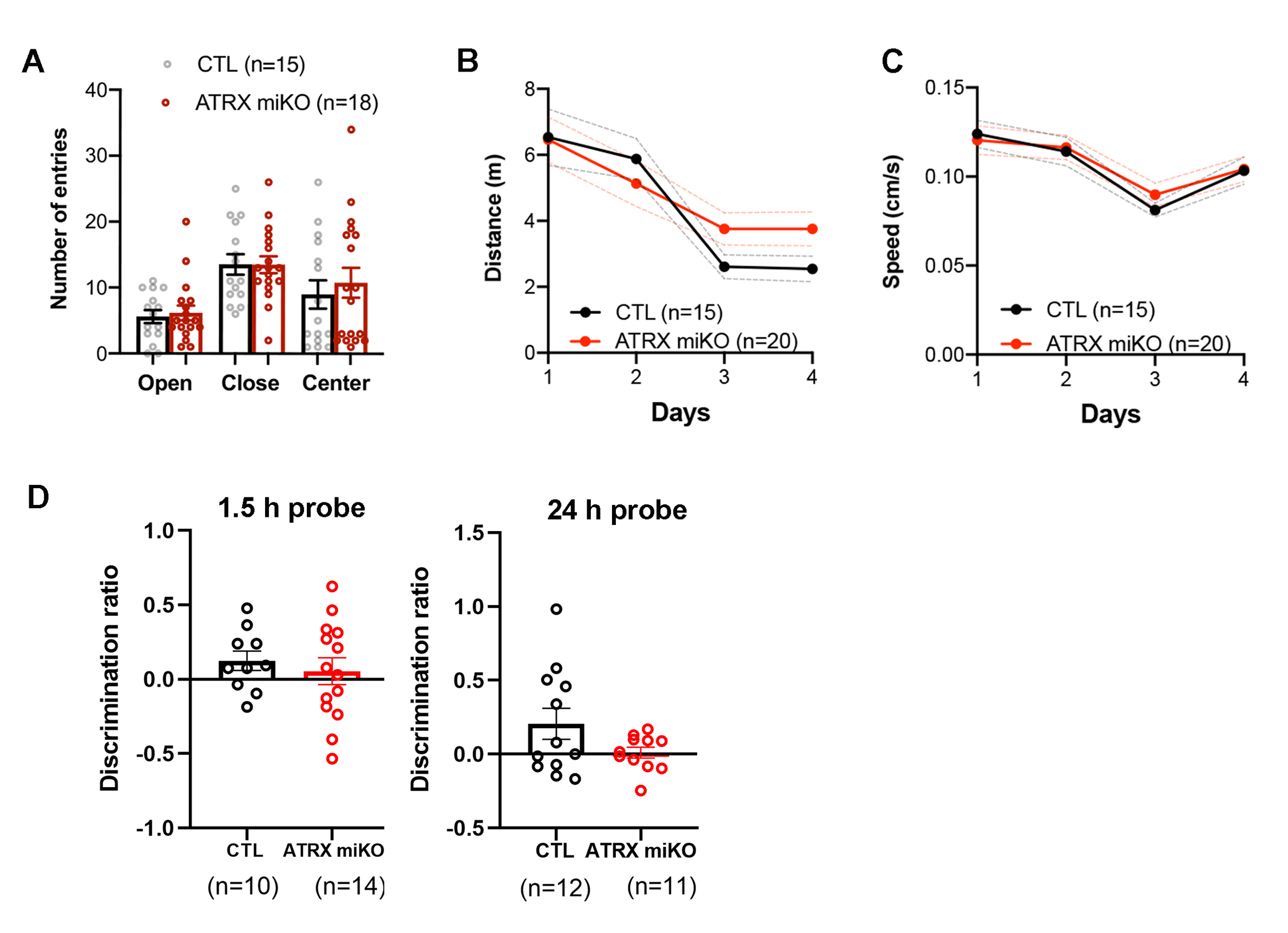

Supplement: S6 Fig — (A) The number of entries in the open and closed arms or in the center of the elevated plus maze over 5 min (open arm entries p = 0.710, closed arm entries p = 0.986, center arm entries p = 0.577, Student T test). Error bars represent ± SEM. (B) Distance traveled and (C) swimming speed over 4 days of training (4 trials/day) in the Morris water maze task (distance traveled F(1, 33) = 0.5162, p = 0.477; swimming speed F(1, 132) = 0.1696, p = 0.681, two-way ANOVA). (D) Discrimination index at 1.5 h (t = 0.5848, df = 22, p = 0.5646) and 24 h (t = 1.696, df = 21, p = 0.1048) probe test of the novel object recognition test. The data underlying this figure can be found in the S1 Data file. (TIF) [file pbio.3002659.s006.tif]
