# Supplementary material for: Microglial deficiency in the ATRX chromatin remodeler elicits a viral mimicry immune response that impacts neuronal function and behavior
Source: PLoS Biol. 2025 Sep 12;23(9):e3002659. doi: 10.1371/journal.pbio.3002659 (PMC12445524; doi:10.1371/journal.pbio.3002659)
Supplement: S12 Table — Details of statistical analyses applied for different behavior tests. (DOCX) [file pbio.3002659.s018.docx]

| Fig. | Groups | N | Measurements | Analysis | F | p | Post-hoc |
| --- | --- | --- | --- | --- | --- | --- | --- |
| 6A | CTL  ATRX miKO | 24  27 | Total distance (Open field) | Unpaired  t-test | T(49)=0.1462 | p=0.8844 | - |
| 6B | CTL  ATRX miKO | 24  27 | Total time at centre (Open field) | Unpaired  t-test | T(49)=0.5973 | p=0.5530 | - |
| 6C | CTL  ATRX miKO | 15  20 | % Time in light (Light Dark Box) | Unpaired  t-test | T(33)=2.150 | p=0.0390 | - |
| 6D | CTL  ATRX miKO | 15  18 | Time in Open Arm  (Elevated Plus Maze) | Unpaired  t-test | T(31)=2.249 | p=0.0318 | - |
| 6E | CTL  ATRX miKO  /Time | 15  18 | Time Freezing/Time (Contextual Fear conditioning) | 2way ANOVA | Interaction F (17, 594) = 0.3188  Time F (17, 594) = 8.772  Genotype F (1, 594) = 5.290 | P=0.9961  P<0.0001  P=0.0218 | Sidak’s m.c.  CTL – ATRX miKO through time  None significant |
| 6F | CTL  ATRX miKO  /Time | 15  18 | Time Freezing/Time (Contextual Fear conditioning-Probe) | 2way ANOVA | Interaction F (11, 363) = 0.5760  Time F (6.772, 223.5) = 4.293  Genotype F (1, 33) = 0.6785 | P=0.8483  P=0.0002  P=0.4160 | Sidak’s m.c.  CTL – ATRX miKO through time  None significant |
| 6G | CTL  ATRX miKO | 15  20 | % Alternations  Entries  (Y Maze) | Unpaired  t-test | Talternations(33)=0.02251  Tentries(33)=0.3063 | P=0.9822  P=0.7613 | - |
| 6H | CTL  ATRX miKO  /Time | 15  20 | Latency  (Morris Water Maze) | 2way ANOVA | Interaction F (3, 132) = 0.6160  Day F (3, 132) = 50.28  Genotype F (1, 132) = 0.4290 | P=0.6058 P<0.0001  P=0.5136 | Sidak’s m.c.  CTL – ATRX miKO through time  None significant |
| 6I | CTL  ATRX miKO  /Quadrant | 15  20 | Time in Quadrant  (Morris Water Maze)  Day 5 | 2way ANOVA | Interaction F (1, 66) = 2.929  Quadrant F (1, 66) = 63.32  Genotype F (1, 66) = 0.8057 | P=0.0917  P<0.0001  P=0.3727 | Sidak’s m.c.  Target: CTL – ATRX miKO  P=0.1342 |
|  | CTL  ATRX miKO  /Quadrant | 15  20 | Time in Quadrant  (Morris Water Maze)  Day 12 | 2way ANOVA | Interaction F (1, 66) = 5.271  Quadrant F (1, 66) = 42.70  Genotype F (1, 66) = 1.666 | P=0.0249  P<0.0001  P=0.2013 | Sidak’s m.c.  Target: CTL – ATRX miKO  P=0.0270 |
| 6J | CTL  ATRX miKO  /Exploration | 22  25 | % Time of exploration  (Novel object Recognition) Training | 2way ANOVA | Interaction F (1, 90) = 5.060e-5  Object F (1, 90) = 0.6158  Genotype F (1, 90) = 0.000 | P=0.9943  P=0.4347  P>0.9999 | Sidak’s m.c.  A-A CTL – ATRX miKO  None significant |
| 6K | CTL  ATRX miKO  /Exploration | 10  14 | % Time of exploration  (Novel object Recognition)  1.5 h probe | 2way ANOVA | Interaction F (1, 44) = 0.6840  Object F (1, 44) = 4.437  Genotype F (1, 44) = 0.000 | P=0.4127  P=0.0409  P>0.9999 | Sidak’s m.c.  A-B CTL, p=0.1188  A-B ATRX miKO , p=0.5472 |
|  | CTL  ATRX miKO  /Exploration | 12  11 | % Time of exploration  (Novel object Recognition)  24 h probe | 2way ANOVA | Interaction F (1, 42) = 5.606  Object F (1, 42) = 7.367  Genotype F (1, 42) = 4.248e-5 | P=0.0226  P=0.0096  P=0.9948 | Sidak’s m.c.  A-B CTL, p=0.0013  A-B ATRX miKO , p=0.9645 |
| S6A | CTL  ATRX miKO  /Arm | 15  18 | Entries  (Elevated plus maze) | 2way ANOVA | Interaction F (2, 93) = 0.1570  Arm F (2, 93) = 10.76  Genotype F (1, 93) = 0.3284 | P=0.8549  P<0.0001  P=0.5680 | Sidak’s m.c.  CTL – ATRX miKO for each area. Not significant |
| S6B | CTL  ATRX miKO  /Time | 15  20 | Distance  (Morris Water Maze) | 2way ANOVA | Interaction F (3, 99) = 1.498  Time F (2.212, 72.98) = 18.57  Genotype F (1, 33) = 0.5162 | P=0.2198  P<0.0001  P=0.4775 | Sidak’s m.c.  CTL – ATRX miKO through time. Not significant |
| S6C | CTL  ATRX miKO  /Time | 15  20 | Speed  (Morris Water Maze) | 2way ANOVA | Interaction F (3, 99) = 0.2885  Time F (2.715, 89.60) = 11.92  Genotype F (1, 33) = 0.1108 | P=0.8336  P<0.0001  P=0.7414 | Sidak’s m.c.  CTL – ATRX miKO through time. Not significant |
| S6D | CTL  ATRX miKO | 10  14 | Discrimination ratio  (Novel object Recognition)  1.5 h probe | Unpaired  t-test | T(33)=0.5848 | p=0.5646 | - |
|  | CTL  ATRX miKO | 12  11 | Discrimination ratio  (Novel object Recognition)  24 h probe | Unpaired  t-test | T(21)=1.696 | P=0.1048 | - |

m.c., multiple comparisons test
