# Supplementary material for: Microglial deficiency in the ATRX chromatin remodeler elicits a viral mimicry immune response that impacts neuronal function and behavior
Source: PLoS Biol. 2025 Sep 12;23(9):e3002659. doi: 10.1371/journal.pbio.3002659 (PMC12445524; doi:10.1371/journal.pbio.3002659)

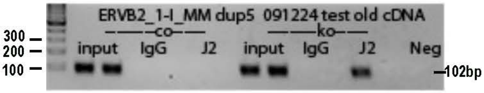

Related to Fig 4J. Agarose gel

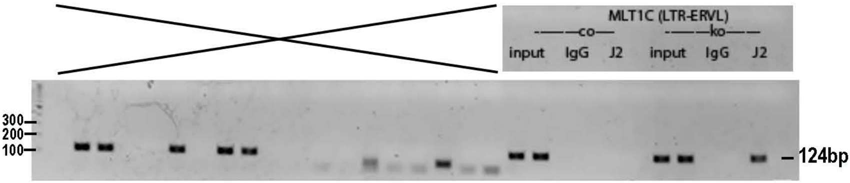

Related to Fig 4J. Agarose gel

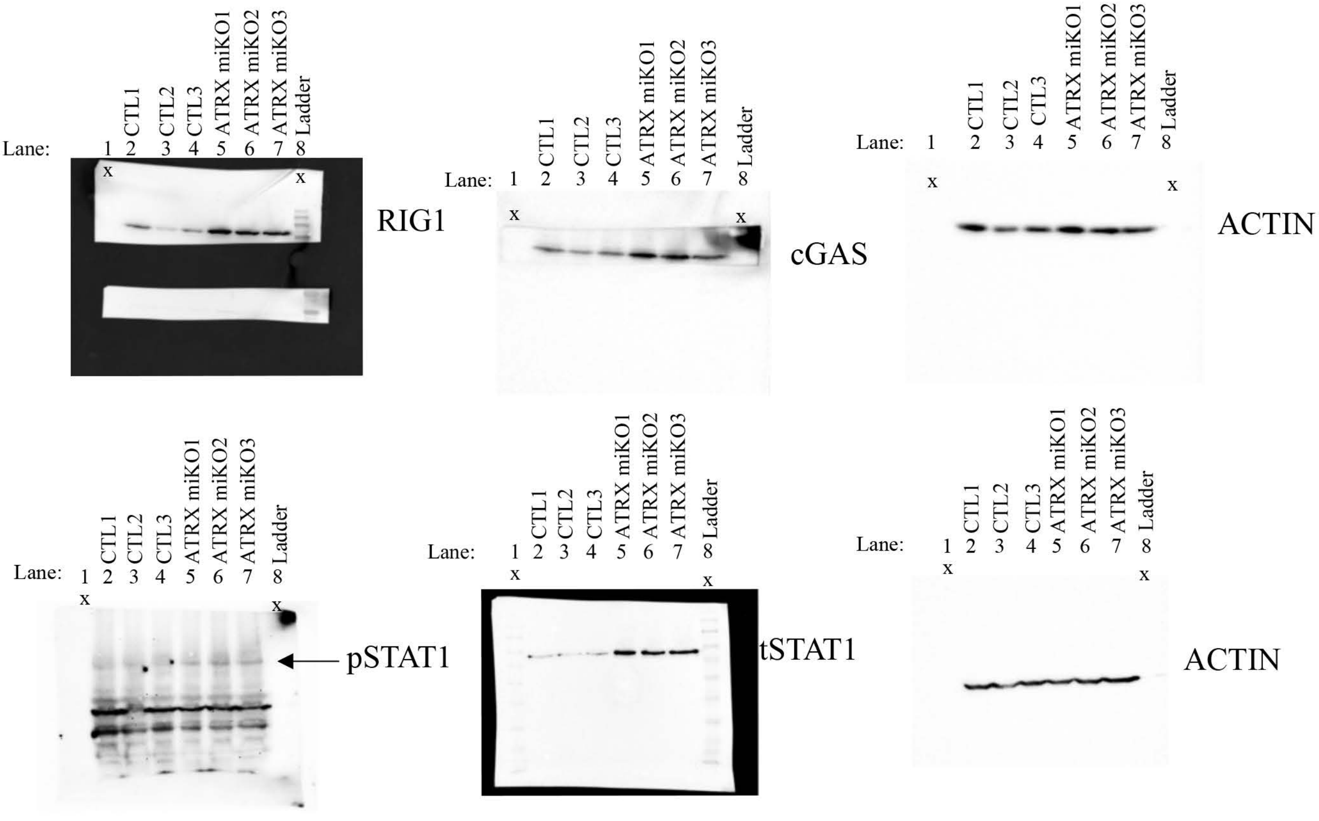

Related to Fig 4K, Captured with chemiluminescent solution (Thermo Fisher Cat# 34095) and exposed using the Universal Hood III (BioRad Cat# 731BR00882).

Figure S4B

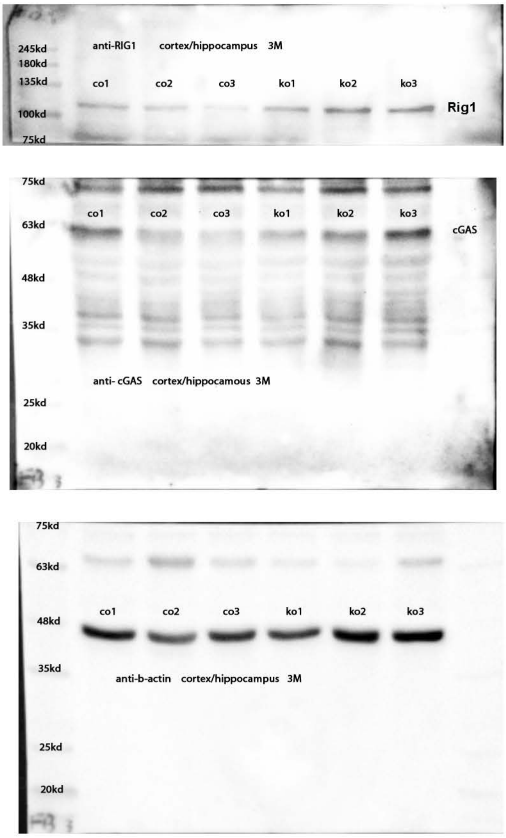

Figure S4C

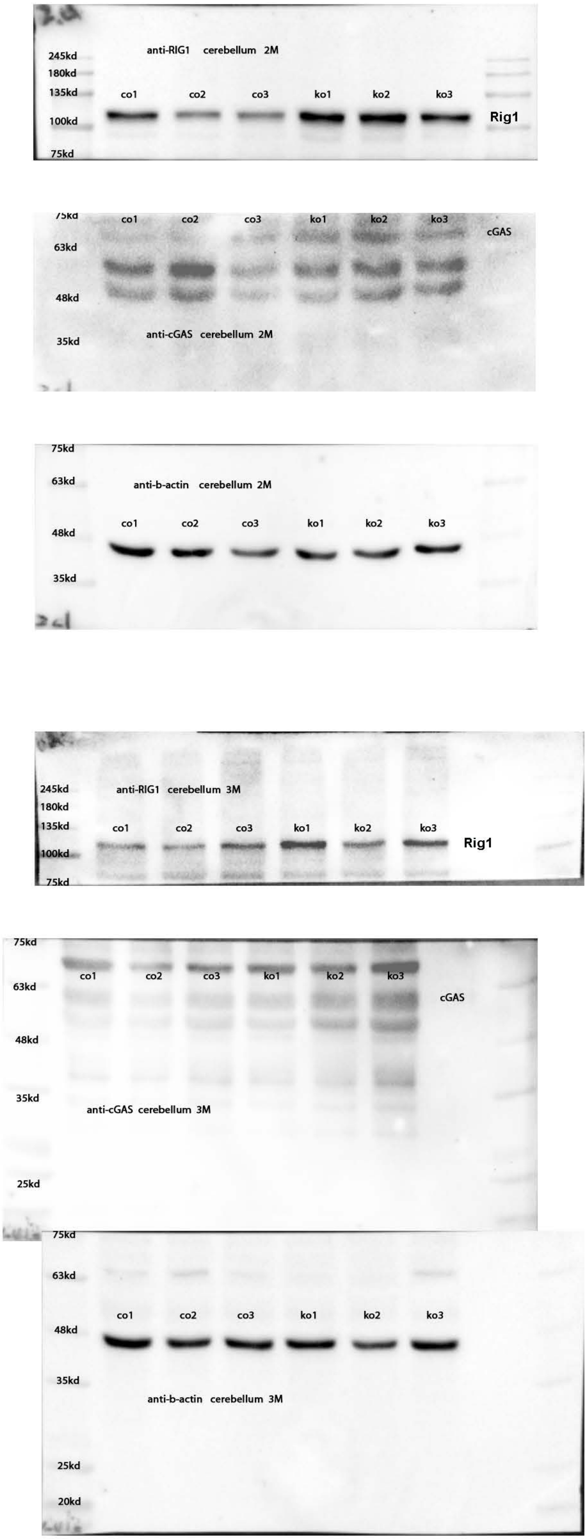

Supplement: S1 Raw Images — File containing raw images for the corresponding cropped images in Figs 4 and S4. (PDF) [file pbio.3002659.s021.pdf]
