# Supplementary material for: Microglial deficiency in the ATRX chromatin remodeler elicits a viral mimicry immune response that impacts neuronal function and behavior
Source: PLoS Biol. 2025 Sep 12;23(9):e3002659. doi: 10.1371/journal.pbio.3002659 (PMC12445524; doi:10.1371/journal.pbio.3002659)
Supplement: Attachment — Submitted filename: Response to reviewers_April21.pdf [file pbio.3002659.s022.pdf]

Response to reviewers

Reviewer #1:

1. I would like to recommend the authors to revisit this analysis, perhaps with help from someone who is an expert in the field.

Response:

Our initial approach (based on Zhang et al, Nature, 2021) consisted of individual TE quantification with RepeatMaster and FeatureCounts (Rsubread) to quantify reads mapping to individual TEs, allowing multiple overlaps and including reads with multiple mapping locations. In this approach, DESeq2 identified ~30,000 individual TEs significantly differentially expressed (padj < 0.05). We now also include a second approach that quantifies families of TE transcripts, according to Jönsson et al, EMBO J, 2021. TEtranscripts (multi-mode) probabilistically assigns multi-mapping reads to TE subfamilies, boosting sensitivity for repetitive elements. This approach, combined with curated TE annotations, led to the detection of ~1,000 expressed TE subfamilies, 40 of which were significantly upregulated in KO samples (mostly LTRs).

In the revised manuscript, we include the results from both analyses (Figure 4D,E), as they complement each other. TEtranscrips specifically addresses the challenge of multi-mapping reads by distributing them probabilistically, complementing the more direct featureCounts strategy that tallies each locus. It also focuses on curated TE annotations (excluding simple repeats, rRNA, tRNA, etc.), reducing noise; featureCounts provides a broader, per-locus view. Both methods reveal a consistent upregulation of LTR-type retrotransposons in ATRX-null microglia, strengthening our initial conclusions by demonstrating reproducibility using different pipelines.

2. For example, the authors do not make a distinction between proviruses and solo LTRs, which is crucial for the suggestion of an ERV-induced viral mimicry. This distinction would greatly improve the precision of the study's conclusions.

Response: From our significantly altered TEs, we extracted the subset corresponding to LTR elements and then categorized them into "Solo LTR" and "Full-Length" groups. To make this distinction, we used the TE naming convention: TE names that include "-int" (e.g., *ERVB2_1-I_MM-int:ERVK:LTR*) indicate the presence of an internal region, suggesting a full-length element. Using this criterion, out of 4,450 significant LTRs, 613 were classified as full-length, and 3,837 as solo LTRs. This information is now mentioned in the main text (page 9, lines 190-192) and the lists are appended that indicate the length of each LTR in Table S6. As expected, the full-length elements are generally longer, with an average length of 1,388 bp, compared to solo LTRs, which average 351 bp.

3. They highlight three LTR families, but the LINE and SINE classes are presented as whole classes (classes that are equally or even more diverse than LTRs). While some of their subfamilies are evolutionary young, the vast majority of the elements in these classes are degenerated copies—therefore, it is crucial to be more specific about which subfamilies are found to be upregulated and how intact these individual elements are. As it is now, all their observations could be based on noise.

Response: We provide additional information on the subtypes of LINE and SINEs that are altered at the transcript and chromatin accessibility level in ATRX-null microglia (Figure 4B,D). We did not previously make this distinction since both intact and degenerate LINEs or SINEs can trigger an innate immune

response. Some degenerate LINEs or SINEs retain promoter activity and can be transcribed into RNA. If these RNAs form dsRNA structures or are aberrantly processed, they can activate RNA sensors such as RIG-I and MDA5, like intact LINE and SINE elements. They can also contribute to genomic instability. DNA damage and the formation of cytoplasmic DNA fragments during repair processes can activate DNA sensors such as cGAS and induce the innate immune response.

4. Figure 4D looks strange - is a threshold missing from the title/legend? How is it possible that NO elements are left unchanged? The same question about the ATAC-seq results and Figure S2D. The authors should reassure the reader that this is not noise. One could show a more global analysis to prove this is not an artefact, for example, with housekeeping genes or describing the ATAC-seq peaks that do not show an effect.

Response: We apologize for the omission. There was indeed a threshold applied to these graphs, therefore the most significantly altered elements are shown. In Figure 4B,D, we exclusively displayed TEs with padj < 0.05. We have updated the figure legendto clarify that only significantly changed TEs are shown. To demonstrate that numerous repetitive elements remain unchanged, volcano plots (and/or global analyses) featuring all data are included, with non-significant points in gray (Figure 4A,C).

5. In addition, the authors claim that the immune response is triggered by the formation of double stranded RNAs derived from TEs. I don't think this is shown by the Strand-specific analysis of the RNA-seq data shown in Figure 4G as they say. Additional experiments are needed to prove the presence of dsRNAs in microglia, as well as more specific characterization of the type of TEs from which these could be transcribed

Response: To demonstrate the presence of dsRNA in the cytoplasm, we stained control and ATRX miKO -derived primary cultures with the J2 antibody that is highly specific for dsRNA and observed increased staining in the cytoplasm of ATRX-null microglia (Figure 4I). We then identified the top upregulated TE sequences that are more likely to form double stranded structures. We pulled down cytoplasmic dsRNA using a J2-coated affinity column from control and miATRX KO brain and performed RT-PCR with top candidates. We identified two upregulated TEs (ERVB2_1-I MMdup5 and MLT1C_LTR-ERVL) that are present in the cytoplasmic fractions as dsRNA in ATRX miKO but not controls (Figure 4J). ERVB2 is evolutionary younger and has a higher potential to directly induce a strong viral mimicry response due to possible transcription of longer, complementary RNA sequences. Text was added to describe these results (Page 10, lines 208-217).

6. In addition, the authors could perform immunostainings for retroviral proteins (e.g. IAP-gag, if these subfamilies are indeed the ones upregulated in the ERVK family).

Response: Unfortunately we were not able to obtain an antibody that worked well.

7. The SINE and LINE examples shown in Figure 4G look like noise or long non-coding RNAs. I encourage them to show a zoom-out with the transcription start site, include the subfamily they are referring to, length, and evolutionary age / conservation.

Response: We provide wider views in Figure S2D.

8. Finally, the authors should discuss the similarities, but also stark differences between mice and human TE composition.

Response: We added to the discussion as follows (Page 16, lines 356-366): Drawing conclusions from these results in mice about associated human disease should be approached with caution given the

differences of retroelements. While retrotransposons are abundant in both mouse and human genomes, comprising approximately 40% of each, there are significant differences in their activity and impact that should be considered. For example, LTR retrotransposons show higher activity in mice and MaLRs are highly active in mice, while ERV1 elements are more prominent in humans. In addition, L1 elements are more active and abundant in mice - there are an estimated 3,000 potentially active L1s in a diploid mouse genome, whereas humans have only about 100 active L1s. The higher retrotransposition rates in mice may lead to more rapid genomic changes, potentially affecting disease progression differently than in humans (70). Despite the differences in TE landscapes, their de-repression can activate an innate immune response in both mouse and human cells.

Reviewer #2:

1. The authors should rephrase the framing of "activated microglia" throughout the manuscript. Our understanding of what makes microglia activated and how this contributes to disease has matured significantly and has become more nuanced. For example, many of these so-called "activation" markers are expressed in cells exhibiting protective phenotypes as well as cells that have become senescent in some states. Defining a binary of activated or not is likely not accurate.

Response: We agree that the use of "activated microglia" is problematic given the complexity of microglial states. We have revised the text, for example referring to an "altered state" "reactive" or "non-homeostatic state" of ATRX-null microglia.

2. There is a peculiar loss of CD68 per cell between 2 and 3 months of age. This hasn't been assessed statistically (but should be). This raises concern about the method being used given that CD68 generally increases with age. The authors should account for why there is this difference, if significant, as well as the decrease in microglial density of microglia (in hippocampus). Does this trend continue in later life?

Response: Thank you for noticing this discrepancy. We initially thought that this occurred because two different individuals performed counts at 2 months vs 3 months. All the images were re-counted by one person, but similar results were obtained. The only possible explanation we can think of is that baseline CD68 is higher in controls at 2 months due to recent tamoxifen injections and that this effect has resolved by 3 months. We extended the analysis to 6 months and see higher Cd68 staining compared to 3 month controls, as expected.

Moreover, the decrease microglial density in Fig S1 is represented as a violin plot whereas all other data are simple bar scatterplots. It's unclear whether this is an example of pseudoreplication where all cells in the groups were compared to one another or whether averages of cell counts in sections for each mouse were compared as appropriate. This should be clarified.

Response: We have revised this and provide bar graphs instead in Figure S1. The results show averages of cell counts in sections for each mouse.

3. The authors should examine which genes among their DEGs presented for highlighted pathways are those that have been previously characterized in the literature to change in response to microglial isolation procedures like FANS. Deletion of ATRX could intersect with these artifactual changes in response to isolation and affect the pathways highlighted.

Response: Given that both control and ko microglia underwent the FANS protocol simultaneously, we make the assumption that any effects on gene expression caused by the method itself would occur in both control and ko cells, and would not appear as differentially expressed. However, it is possible that ATRX-null microglia are more or less sensitive to the FANs procedure and would react differently at the

transcriptional level than control cells. However, such an ATRX-mediated effect would not be observed in other FANS studies. Note also that the method we used bypasses several problematic artefacts encountered by other approaches since there is no cell dissociation steps that rupture cell processes, and the nuclei are already fixed during the sorting step.

4. The choice of 2 vs 3 months for ages of study should be justified. No rationale for this is given, and it's unclear what phenotypes would be expected at developmental timepoints and later age timepoints based on the results shown. As the introduction highlights the importance of this gene for intellectual disability, it seems necessary to justify ages and why deletion wouldn't make sense at an earlier timepoint prior to adulthood (2 and 3 months).

Response: The time points were chosen to capture short and longer term effects of ATRX inactivation in microglia. Tamoxifen was administered at P45-P50 and we wanted to examine the outcome relatively shortly after (10 days later, at P60) and again a month later (P90) to see if initial effects are maintained or transient. This approach indeed revealed differences shortly after inactivation vs a month later (i.e. increased proliferation). We agree that given ATRX's role in a developmental disorder that it would make sense to induce ATRX deletion earlier than P45. We have done an early postnatal tamoxifen treatment of lactating dams (P1-P3). At postnatal day 30, we verified the efficiency of ATRX inactivation (>90%) and observed altered microglia morphology and increased CD68 levels. However, the Atrx miKO mice had no detectable behaviour or cognitive deficits. Further investigations indicate that a subset of ATRX-null microglia die at earlier stages with replenishment by ATRX + microglia. As this part of our study is confusing due to compensating mechanisms, we are planning on writing a small report to publish separately rather than adding to the current manuscript.

5. The statement that multiple genes belonging to DNA/RNA sensing pathways are overexpressed in ATRX-null microglia from RNAs-seq (lines 190-191) should be substantiated with enrichment analyses and graphs of the individual genes (for supplement). This is key to the argument being made so "data not shown" is not sufficient evidence.

Response: We added the transcript data for genes in these pathways in Figure S4A.

6. How generalizable is the elevation of cGAS? Is this also true in hippocampus? Is it consistent across ages?

Response: The western blots at 2 months are done on cortex and hippocampus. To determine whether this is sustained, we performed western blots at 3 months and find that RIG1 (but not cGAS) is significantly increased in the KO cortex/hippocampus (Figure S2E). To see if this is similar across different brain regions, we also repeated this in the cerebellum at 2 and 3 months and find that both RIG1 and cGAS protein levels are significantly increased (Figure S2F).

Also, immunoblotting experiments showing elevations of DNA/RNA sensing components (cGAS, RIG-1, STAT1, etc) are all performed in whole cortex tissue, yet the change is presumably only in microglia, one portion of the total pool in a lysate. Does dysfunction in microglia lead to similar changes in neurons, which is driving the effects, or is it specific to the microglia

Response: RIG-I mediated RNA-sensing of endogenous cytoplasmic dsRNAs in microglia can potentially cause activation of this same pathway in other cell types, particularly astrocytes and neurons. Microglia are the primary producers of type I IFNs in response to RNA sensing in the central nervous system. These IFNs can act on other brain cells to enhance their antiviral responses and activate RIG-I pathways. Therefore, ATRX-null microglia could potentially transfer antiviral signals to other cell types through secreted factors. For example, supernatants from activated microglia can induce antiviral states in

neurons in a STING-dependent manner. Microglia-derived factors can upregulate TLR3 expression in astrocytes, priming them for enhanced responses to RNA stimuli. This intercellular signaling network allows microglia to amplify antiviral and inflammatory responses across different cell types in the brain, creating a more coordinated defense against both pathogenic and potentially harmful endogenous RNA species.

How do isolated microglia look for these components?

Response: The RNA-seq data obtained from sorted microglia nuclei show increased expression of many genes in that pathway (Figure S4A).

7. Several interpretations are often heavy-handed. For example, the authors link cytokine elevations to the antiviral immune response and cGAS the identified, but many pathways they reveal from pathways analysis of DEGs imply that many causes may lead to these changes. Moreover, the authors have not shown whether this deletion causes astrocytosis as a consequence, which could also lead to increases in cytokines.

Response: We showed that several cytokines are upregulated in the purified microglia nuclei (from RNA-seq data), so part of the cytokine increase comes from ATRX-null microglia, but it could also be amplified in astrocytes. We have modified the text to point out the possibility that the upregulation of cytokines observed could also be the result of astrocytosis (Page11, line243-244).

8. The issue in point #7 occurs also in the discussion when the authors argue that the cognitive dysfunction likely stems from the cytokine elevations when they have shown data that neuronal excitability is somehow affected (which likely also affects cognition). Moreover, the discussion fails to provide a satisfying explanation for how microglial deletion of ATRX affects neuronal excitability.

Response: Cytokines are only one possible cause of cognitive deficits. We modified the discussion to clarify that cytokine production could affect other cell types, including neurons and that these neuronal changes would in turn lead to cognitive changes (Page 18 line 392-398). There is limited knowledge on how cytokine production can affect neuronal excitability, although there is evidence that it leads to upregulation of sodium current densities in neurons. Alternatively, cytokines can directly interact with neuronal receptors. For example, IL-1 may increase neuronal excitability through direct interaction of its receptor complex with NMDA receptors. Alternatively, cytokines could induce transcriptional changes in neurons, altering the expression of ion channels and receptors that regulate excitability. We added data on the effect of ATRX-null microglia on hippocampal CA1 neuronal morphology, showing increased branching and increased filopodia spines (Figure 5A-C). This could potentially be explained by hyper-ramification of ATRX-null microglia in this region, resulting in increased microglia-dendrite interactions and inducing filopodia formation (71). Alternatively, microglia lacking ATRX might fail to eliminate "weaker signals" such as filopodia spines (72).

9. What is the basis on which each of the subclusters is annotated? Can deconvolution be a robust method here to create subclusters if the KO creates significant shifts in microglial state that leads to differing populations not present in the original single cell dataset?

Response: The subclusters are annotated based on the original single-cell dataset. We then evaluated the enrichment of each subcluster by comparing the gene expression profiles between the knockout and control samples. This approach can only detect microglial states that were present in the original dataset. It is a limitation of this particular approach that we now acknowledge in the revised manuscript (Page 7, lines 148-149).

10. The authors make a statement that all data underlying the manuscript are available in the supporting documents and reporting checklist and not upon request. Therefore, it is confusing that there are multiple instances of "data not shown" throughout the manuscript. As one example, one of the key issues is whether other cell types are affected by microglial deletion of ATRX (and how), yet this is not addressed aside from a statement that they looked at astrocytes and oligodendrocytes, which is not shown.

Response: We have gone through the manuscript and identified the instances where this occurred and have now provided the data. We also include the data showing the limited upregulation of TEs in sorted ATRX-null astrocyte and oligodendrocyte nuclei compared to ATRX-null microglia nuclei (Figure S3, Table S8). We also now show the transcriptional changes in the DNA and RNA-sensing pathway genes (Figure S4)

11. In Figure 6, J through K should also be shown along with discrimination index. Importantly, there appears to be no multiple comparison testing with post tests. Only t tests appear to be used here, which is not correct. This should be corrected throughout the manuscript where it occurs.

Response: We added the discrimination index for Figure 6 J-K in Figure S6D. We also repeated the statistical analysis with multiple comparison testing. This reveals a memory deficit at 24hrs, but not at 1.5 hr.

Reviewer #3:

I find that this manuscript reads well, the findings are presented with clarity, the methods used are broad (including RNAseq of FACS-isolated nuclei, ATAQ-seq, electrophysiology and behaviour testing) and adequate, and the experimental data mostly support the conclusions made by the authors. Their data also further reinforces the emergent view that microglia are key regulators of synaptic function and that microglia dysfunction might play a central roles in the onset and/or progression of different neurological disorders.

I have only a few minor points:

1) The categorization of microglia shape suggests changes in microglia reactivity triggered by the loss of ATRX. Together with the CD68 IHC and transcriptomic data the authors conclude that microglia are reactive. It maybe so, however the quality of the microglia morphological characterization does not match the general quality of the paper. The authors should consider in illustrating such changes in microglia morphology with good quality and higher magnification representative pictures (for example in Fig1) . In addition, Scholl analyses of microglia would provide a much better (and quantitative) assessment of changes in microglia morphology.

Response: We agree that the assessment of microglia morphology was unsatisfactory and we now provide a more extensive evaluation that includes representative tracings and more sophisticated analysis. This revealed that in addition to larger and more rod-shaped somas and nuclei, ATRX-null microglia have hyper-ramified processes with increased complexity of branching at the periphery (Figure 1E-I). Indeed, IFN-α-activated microglia exhibit larger cell bodies and hyper-ramified processes (as opposed to IL-6, for example), challenging the notion that all activation states reduce branching.

Ref: West PK, McCorkindale AN, Guennewig B, Ashhurst TM, Viengkhou B, Hayashida E, Jung SR, Butovsky O, Campbell IL, Hofer MJ. The cytokines interleukin-6 and interferon-α induce distinct microglia phenotypes. J Neuroinflammation. 2022 Apr 16;19(1):96. doi: 10.1186/s12974-022-02441-x. PMID: 35429976; PMCID: PMC9013466.

2) In Figure1 E in the Y axis - correct "elogated" to elongated

Response: This was corrected

3) In Figure 2 F is difficult (at least in my PDF) to really identify Ki67 + cells. Also the clear difference in proliferation shown in the graphic between control and ATRX miKO at 2 months is not evident in the figure. An inset with higher magnification would help.

Response: We provide better images of Ki67 and insets with higher magnification.
