# Supplementary material for: Microglial deficiency in the ATRX chromatin remodeler elicits a viral mimicry immune response that impacts neuronal function and behavior
Source: PLoS Biol. 2025 Sep 12;23(9):e3002659. doi: 10.1371/journal.pbio.3002659 (PMC12445524; doi:10.1371/journal.pbio.3002659)
Supplement: Attachment — Submitted filename: Response to reviewers_August11_2025.pdf [file pbio.3002659.s024.pdf]

Response to reviewers (August 11 2025)

Reviewer #1, Johan Jakobsson and Raquel Garza (note, Drs Jakobsson and Garza reviewed this study together, and provided comments as reviewer 1. They have opted to sign this review:

Overall, the comments regarding ERV classification and interpretation of the results are much better and the manuscript is now ready for publication. However, a couple of details that could help the reader are still missing:

1. The methods section described the stringency of the mapping of the reads for the multi-mapping for TEtranscripts quantification, but the authors haven't specified the parameters used for the mapping that was used for the per-element differential expression analysis or, e.g. the tracks in Figure 4H.

Response: We have revised the methods section to specify that, for per-element TE quantification, reads were aligned with HISAT2 (RNA-seq) or Bowtie2 (ATAC-seq) using default parameters. Only uniquely mapping, non-duplicate reads were counted in featureCounts (countMultiMappingReads = FALSE; --ignoreDup). These details, along with average mapping rates and a complete sample-level breakdown of read assignment categories, are now provided in the revised methods (Lines 619-625) and Table S13.

2. This is equally or more important for the ATACseq. In general, it would be good for the authors to give some details on the mappability of the reads to these elements for both of the sequencing technologies.

Response: We added detailed mappability information for RNA-seq and ATAC-seq in the revised manuscript. The methods section now specifies that on average, 25% of uniquely mapped reads were assigned to repeat elements in both assays. Table S13 provides full counts for all read assignment categories (assigned, duplicate, no feature, ambiguous, unmapped) for each sample.

3. "LTR-type" should be rephrased to "LTR class" (same with LINE and SINE type —> class). Assuming that the figure is indeed showing LTR families

Response: We have made these changes in the text (Lines 193 and 197)

4. "In contrast, most human ERVs are highly mutated and lack retrotranspositional capacity" No HERV has been proven to retrotranspose in the human genome.

Response: We appreciate this comment and agree that, to date, no endogenous HERV has been shown to retrotranspose in the human genome under physiological conditions. Our use of the qualifier "most" was intended to reflect findings from experimental models. We have changed the text to: "In contrast, human ERVs are highly mutated and lack retrotranspositional capacity *in vivo*, which likely limits their direct immunogenic potential compared to their murine counterpart (72)." (Lines 373-375)

Reviewer #2:

Overall, the authors have sufficiently addressed concerns for all rounds of review. The only lingering issue relates to the interpretation of hippocampal-dependent memory in the manuscript. While the authors acknowledge that there are no true changes in NOR data in the mice that reached statistically significance to warrant claims that the mice have functional differences, the authors show a significant difference in MWM data on the 12th day of testing that does reach significance. Overall, the spatial memory results in the manuscript are not sufficiently robust to make the claim there is a change in hippocampal spatial memory. Multiple assays would be needed to make this claim, and showing a difference at one timepoint in one assay does not support this claim overall. This was adequately addressed in the text, but the limitation should be addressed in the title by removing the reference to memory.

Response: We changed the title to "Microglial deficiency in the ATRX chromatin remodeler elicits a viral mimicry immune response that impacts neuronal function and behavior".
