# Supplementary material for: Microglial deficiency in the ATRX chromatin remodeler elicits a viral mimicry immune response that impacts neuronal function and behavior
Source: PLoS Biol. 2025 Sep 12;23(9):e3002659. doi: 10.1371/journal.pbio.3002659 (PMC12445524; doi:10.1371/journal.pbio.3002659)
Supplement: Attachment — Submitted filename: Response to reviewers_June2.docx [file pbio.3002659.s023.docx]

**Response to reviewers:**

**Reviewer 1:**

***1. An internal region does not necessarily mean an ERV is a provirus or "full-length." Tools like Retrotector (Jönsson et al., 2021) can better classify ERVs by stitching together fragmented internal regions and LTRs. The analysis of full-length ERVs should be updated for accurate interpretation.***

**Response:** Thank you for this suggestion. To assess whether each upregulated LTR was a solo LTR or part of a full-length or partial provirus, we extended each LTR region by ±15kb in the reference genome. This extension provides the necessary genomic context to detect the presence of potential flanking LTRs and internal retroviral genes (gag, pol, env) that define full-length ERVs, as opposed to truncated or solo LTRs. We then used RetroTector to classify each locus according to the completeness of retroviral structural motifs. An element was classified as a full-length provirus if all major internal retroviral domains were detected along with both flanking LTRs. Loci missing one or more of these domains but still containing substantial internal retroviral features were classified as partial proviruses. LTRs lacking significant internal retroviral domains were annotated as solo LTRs.

Of the 3,202 upregulated LTRs analyzed, RetroTector identified 99 as full-length proviruses, 462 as partial proviruses and the remaining 2,641 as solo LTRs. We updated the text to include this information. (Page 9, lines 194-196). We also updated the materials and methods section to reflect this new analysis (Page 29, lines 640-657)

***2. Provide the relevant scripts in a well-documented GitHub repository, ideally with a Zenodo DOI.***

**Response:** We have done this and added the following text in the main manuscript (Page 38, lines 843-847). “All relevant analysis scripts and pipelines used for RNA-seq, ATAC-seq, and ERV classification are available in a publicly accessible GitHub repository: <https://github.com/aliireza96/Shafiq_PlosBiol_ATRX_microglia>. The repository includes detailed documentation and instructions to reproduce all computational analyses. A snapshot of the repository has been archived on Zenodo with DOI: <https://doi.org/10.5281/zenodo.15679297>.”

***3. Expand the discussion of differences between mouse and human TE landscapes. Notably, mice have ERVs that can retrotranspose, while humans do not, which has major consequences for control and activation.***

**Response:** We modified the text in the discussion to emphasize this point (Page 17, lines 368-381).

***4. Figure S1F y-axis label is overlapping the numbers.***

**Response:** This was rectified, thank you.

***5. Figure 4B,D should show the number of elements being up/down regulated (and preferably the total. E.g. 20/1000)***

**Response:** We added the number of elements displaying significantly increased or decreased accessibility in the figure legend of Figure 4B. We also include the number of element transcripts up- and down-regulated in the figure legend of Figure 4D.

***6. Figure 4H has an illustrator text box showing "Lorem ipsum".***

**Response:** Thank you, this was removed from the figure.

**Reviewer 2**

***1. In many cases, legitimate concerns have been raised and then addressed thoroughly in the rebuttal text, but these responses have not been described in the discussion or in supplementary data where they could benefit the manuscript in a substantial way. See examples for the second part of point #6, etc and other examples throughout responses to previous concerns.***

**Response:** We have integrated key elements from our previous rebuttal directly into the main text as requested.

(1) Regarding the concerns about non-specific transcriptional effects of FANS, we added “Given that both control and ATRX miKO undergo FANS simultaneously, we assume that any effects on gene expression caused by the method itself would occur in cells of both genotypes and would not appear as differentially expressed. The method also bypasses several problematic artefacts: It doesn’t include cell dissociation steps that rupture cell processes, or nuclei staining that could affect chromatin structure.” (Page 6, Lines 123-128)
(2) Regarding effects of microglia reactivity on other cells, we added: “Microglia function as the central hubs for innate immune signaling in the brain, initiating a cascade that extends to other cell types. The activation of RIG-I by endogenous dsRNAs in microglia leads to the secretion of type I interferons, which act as potent paracrine signals. These IFNs can engage receptors on nearby astrocytes and neurons, enhancing their antiviral capabilities by activating their own RIG-I pathways. This intercellular signaling network may allow ATRX-null microglia to amplify antiviral and inflammatory responses across different cell types in the brain, creating a more coordinated defense against potentially harmful endogenous RNA species (Page 19, lines 419-434)
(3) Regarding altered microglia morphology/reactivity, we added: “Our findings show that ATRX is required cell-autonomously in microglia to maintain homeostasis. In addition to larger and more rod-shaped somas and nuclei, ATRX-null microglia have hyper-ramified processes with increased complexity of branching towards the periphery. This resembles IFN-α-activated microglia and challenges the notion that all activation states reduce branching.” (Page 16, lines 349-353)

***2a. In Figure 6, several behavioral analyses (6I and 6k) are showing differences in performance only within a genotype but inferring differences across genotypes based on lack of difference within one of the genotypes. This type of "implicit" comparison as an approach is a statistical error and lacks rigor, as described in detail previously (***[***https://www.nature.com/articles/nn.2886***](https://www.nature.com/articles/nn.2886)***).***

**Response: We have revised our statistical approach for the data shown in Figure 6I (Morris Water maze probe tests) and 6K (Novel object recognition probe tests).**

**6I: Two-way ANOVA reveals a significant interaction for the Morris water maze probe test at day 12 (long-term spatial memory), but not at day 5.**

**6K: Similarly, we detect a significant interaction in the Novel object recognition test, but the discrimination ratio is not significantly different. Consequently, we have revised the text to reflect that we observe spatial memory deficit and the title (“hippocampal-dependent memory”) but acknowledge that the effect of ATRX loss on novel object recognition memory is modest. For clarity, we also include a supplementary table listing the results of statistical analyses for all the behaviour tests (Table S12).**

***2b. Also, why is a Violin plot provided for 6C and D? Are these showing individual mouse replicates or comparisons across multiple trials? The former should be shown as a scatterplot.***

**Response:** The **Violin plots in Figure 6C and 6D have been replaced with scatterplots to represent individual mouse replicates.**

***3. Somewhat related to the previous point, but the behavioral results overall are somewhat problematic in how they're being interpreted - there is not solid evidence that there are cognitive effects of sufficient quality to argue functional changes here. After requesting the discrimination index to be reported in Figure 6, this has now been included, though it is not commented upon that the effect is not significant, supporting the overall trend that there are not robust cognitive changes following manipulation of ATRX. The question raised now is whether the mechanism put forth by the authors requires cognitive changes for it to be valid. Overall, the behavioral data argue that the effect of ATRX modulation is not strongly linked to cognitive changes. This has not been placed in the context of the model being proposed in the paper.***

**Response:** We agree with the reviewer's assessment of the Novel Object Recognition (NOR) results. Upon re-evaluation, although we detect a significant interaction from two-way ANOVA, suggesting a deficit, the difference in the Discrimination Index between genotypes is not significant, and we have therefore toned down this claim in the manuscript.

This stands in contrast to the deficit we observed in the Morris Water Maze (MWM). The day 12 probe trial revealed a significant genotype-by-quadrant interaction, providing evidence of impaired long-term spatial memory in the mutant mice.

We have modified the text to accurately reflect these findings, framing the cognitive phenotype as one primarily restricted to a spatial memory deficit, which is well-supported by the MWM data.
